# A multidimensional analysis of older adults wellbeing and health literacy in Alentejo: a cross-sectional study

**DOI:** 10.3389/fpubh.2025.1514968

**Published:** 2025-04-25

**Authors:** Rute Sadio, Adriana Henriques, Paulo Nogueira, Andreia Costa

**Affiliations:** ^1^Nursing Research, Innovation and Development Centre of Lisbon (CIDNUR), Nursing School of Lisbon (ESEL), Lisbon, Portugal; ^2^Unidade Local de Saúde do Alentejo Central, Estremoz, Portugal; ^3^Instituto de Saúde Ambiental (ISAMB), Faculty of Medicine, University of Lisbon, Lisbon, Portugal; ^4^Laboratório para a Sustentabilidade do Uso da Terra e dos Serviços dos Ecossistemas – TERRA, Lisbon, Portugal

**Keywords:** quality of life, wellbeing, functionality, loneliness, health literacy

## Abstract

**Introduction:**

The global increase in the older adults population which represents 22% the population in Portugal and is especially pronounced in the Alentejo region, posed noteworthy challenges. Social isolation, particularly in rural areas, requires policies that promote inclusion and wellbeing, such as social prescription. This study aimed to conduct a multidimensional assessment of the older adults individuals in Alentejo, evaluating quality of life, wellbeing, functionality, loneliness and health literacy.

**Methods:**

A cross-sectional design was performed involving 344 participants aged 65 and over, residing in the Alentejo region. Validated scales and Questionnaires were used to assess sociodemographic characteristics, quality of life, wellbeing, functionality, loneliness and health literacy. Data were analyzed using SPSS software, employing descriptive and inferential statistics to identify significant patterns and relationships between variables.

**Results:**

The sample consisted of 52% women, with an average age of 75.3 years (SD = 7.31; range = 65–96 years). Wellbeing, as measured by the WHO-5 index, was preserved with an average score of 53 (SD = 4.29). Loneliness was prevalent, with 50% of participants experiencing it, and the average score on the UCLA Loneliness Scale was 41.9 (SD = 5.59). Pain and anxiety were the most commonly reported issues according to the EQ-5D-3L, with 57.3% reporting pain/discomfort and 48.5% reporting anxiety/depression. Health literacy levels were low, with, only 6.4% exhibiting excellent literacy, while 45.0% had problematic or inadequate literacy.

**Discussion:**

The findings suggest that while preserved, significant differences exist between men and women, as well as between rural and urban residents. Mobility, pain, and anxiety were the primary factors affecting Quality of life, particularly in rural areas. Low health literacy was identified as a barrier to autonomy and effective health management, emphasizing the need for tailored interventions to promote active and healthy aging.

## 1 Introduction

The global population is rapidly aging, with an estimated 727 million people aged 65 or older in 2020, accounting for approximately 9% of the world’s total population. By 2050, this figure is projected to more than double, reaching 1.5 billion (1). In Portugal, the older adults already represents 22% of the population, with significant regional disparities, such as the Alentejo region, where 27% of the population is aged 65 or over, with has 219 older adults individuals for every 100 young ones (2). This demographic shift presents both opportunities and challenges, particularly in regions like Alentejo, which is predominantly rural.

Older adults living in rural areas face unique challenges, including limited access to health services, social support, and transportation. These factors can lead to an increased risk of social isolation, health issues, and reduced quality of life, particularly when compared to their urban counterparts (3). However, rural environments can also offer benefits, such as the presence of green spaces, which promote physical and mental wellbeing, particularly during periods of societal disruption, such as the COVID-19 pandemic (4, 5).

In addition to the implications of aging for public health and health care systems globally (6), the challenges faced in adapting to the effects of population aging are also specific to low-and middle-income countries (7). One of the challenges is the increasing dependency rate in many countries, including Portugal. Authors such as Cheng et al. (8) warn that an increase in the dependency ratio can put a strain on available resources and on ensuring that older people receive the care and support they need. In 2019, the older adults dependency ratio was 34.5 (9).

When we talk about aging, we have to take a multidimensional approach to understanding it, with the aim of achieving an improvement in the quality of life of the older adults person, which is influenced by a series of interconnected factors, ranging from physical and mental aspects to social and environmental issues (10). Quality of life in aging is strongly related to access to adequate financial resources and opportunities for meaningful social involvement (11). In addition, according to studies, quality of life in aging is also linked to maintaining healthy family and social relationships, as well as the ability to adapt to changes in life circumstances. This means that policies and programs aimed at the older adults should consider not only health care, but also access to social services, opportunities for community participation and safe and inclusive environments (8, 12).

As a direct influence on aging, the COVID-19 pandemic has brought to light urgent issues related to aging and the wellbeing of older people. Social distancing measures and mobility restrictions have had a significant impact on older people’s lives, increasing levels of loneliness and social isolation. According to studies conducted by Liu et al. (13), this age group has been particularly affected by the pandemic, facing additional challenges due to factors such as increased risk of health complications and reduced social support. There has been a significant increase in levels of loneliness during the pandemic, especially among older people (14). Loneliness is a public health problem that affects a significant proportion of the older adults population worldwide (15, 16). In addition, loneliness has been identified as a risk factor for premature mortality in the older adults, rivaling smoking and obesity in its magnitude of adverse effects (17). Address this challenge, interventions and policies aimed at mitigating loneliness among older people are key. Community-based approaches, such as home visiting programs, social support groups and volunteer initiatives, have been suggested as effective ways to reduce loneliness among older people (18).

Literacy being interconnected themes that require attention and action, it is fundamental for the wellbeing and quality of life of older people, as it is directly linked to autonomy, social participation and access to health services. In Portugal, the proportion of older people with low levels of literacy is significantly high (19). The older adults population with low literacy levels is known to face additional difficulties in understanding health information, making better decisions about health care, and adhering to treatment instructions and improving their health (20, 21). To deal with this problem, interventions and programs to promote health literacy in the older adults population are essential. This would reduce health costs, improve the relationship and communication between the person and the health professional and meet their needs (22). Community-based approaches, such as literacy courses, reading groups and creative writing activities, have been shown to be effective in improving literacy skills among older people (23). In addition, the use of information and communication technologies, such as tablets and cell phones, can help increase access to information and educational resources (24, 25).

Although previous studies have addressed the wellbeing of older adults, gaps remain in understanding health literacy, loneliness, and functioning in rural settings (17, 26, 27). Furthermore, in Portugal, there is limited evidence on the effectiveness of community interventions specifically adapted to older adults rural populations (28–30), mainly in the Alentejo region, which is the most rural and aging in the country (31).

This study aims to provide a comprehensive assessment of the older adults population in the Alentejo region, focusing on their quality of life, wellbeing, functionality, loneliness, and health literacy. This research seeks to fill these gaps with localized insights to guide healthy and active aging strategies, particularly in rural settings.

## 2 Materials and methods

### 2.1 Design of the study and participants

A community-based cross-sectional study was carried out in order to meet the proposed objectives. This type of study allowed for the collection of data at a point in time on the characteristics and behaviors of the target population, providing important insights for understanding health and behavioral issues. Cross-sectional analyses offer a snapshot of characteristics and associations in a population, allowing an initial and rapid understanding of patterns of health and disease (32). This study investigates the wellbeing, health literacy, and quality of life of older adults individuals in the Alentejo region. The research question is formulated using the PICO framework as follows: P (Population): Older adults individuals aged 65 and over registered in a Personalized Health Care Unit (UCSP) in the Alentejo; I (Intervention/Exposure): Multidimensional assessment involving quality of life, wellbeing, functionality, loneliness, and health literacy; C (Comparison): Differences between rural and urban residents, and between men and women; O (Outcome): Identification of key factors affecting wellbeing, health literacy, and quality of life to inform policy and interventions promoting healthy aging.

The sample was selected in a non-probabilistic way (33), participants were invited to participate by a health professional involved in their care process, based on availability and accessibility, contributing to this data collection process all health professionals from the unit, including a researcher.

The sample size was determined as 344 participants, the sample calculation was calculated with a sampling error of 0.05, taking into account that the prevalence of adherence is approximately 50% and using SurveyMonkey software, including a 20% margin for missing data.

To address the research question effectively, a series of hypotheses were formulated to explore potential differences and associations within the target population. These hypotheses will serve as a foundation for understanding the key determinants of wellbeing and health literacy. H1: Older adults individuals residing in rural areas experience lower wellbeing and quality of life compared to those in urban areas. H2: Women report higher levels of loneliness and lower health literacy compared to men. H3: Low health literacy is associated with reduced functionality and increased anxiety/depression among the older adults population.

Following the hypotheses, the main objectives of this research were outlined, which focus on evaluating the older adults population’s quality of life, wellbeing, and health literacy, while identifying specific areas that require targeted intervention. O1: To assess the quality of life, wellbeing, and health literacy of older adults individuals in Alentejo. O2: To analyze the impact of gender and geographic location (rural vs. urban) on wellbeing, loneliness, and health literacy. O3: To identify the primary factors influencing quality of life, including pain, mobility, and anxiety. O4: To provide recommendations for community-based interventions aimed at promoting active and healthy aging in rural regions.

### 2.2 Inclusion and exclusion criteria

The inclusion and exclusion criteria for this study were designed following the PICO framework to ensure a focused and relevant sample population. The inclusion criteria for this study were older adults individuals aged 65 and over residing in Alentejo, registered with a Personalized Health Care Unit (UCSP). Participants must have the cognitive ability to complete questionnaires, communicate in Portuguese, and have telephone contact available at the units. Participation in multidimensional assessments through validated questionnaires and scales. Exclusion criteria were individuals unable to provide informed consent. Those with severe cognitive impairments preventing the completion of questionnaires. Older adults individuals on waiting lists for National Continuing Care Network facilities or those who did not respond after three attempts to contact them by telephone.

### 2.3 Ethical considerations

The study was approved by the ARSA Ethics Committee (Advice 17/CE/2022), ensuring that it adhered to all ethical guidelines and requirements. Written informed consent was obtained from all participants, and confidentiality was maintained throughout the research process.

### 2.4 Data collection procedure

Data were collected opportunistically as participants attended UCSPs for routine activities, such as nursing appointments, treatments, or vaccinations. The data collection process, which took place between July 2023 and March 2024. Health professionals identified eligible participants and provided them with an explanation of the study’s objectives. Participants who agreed to participate were given informed consent forms and questionnaires. They had the option to complete the questionnaires at the UCSP or at home, with assistance from family members or healthcare professionals as needed.

This method of data collection made it possible to take advantage of the presence of older adults people in the UCSP and ensure that the process of participation was clear, consensual and convenient for the participants.

### 2.5 Instruments

Validated tools were used to measure the key dimensions of wellbeing, quality of life, functionality, loneliness, and health literacy. These included:

**The WHO-5 Wellbeing Index**: Developed by the World Health Organization, this scale assesses subjective wellbeing through five statements rated on a scale of 0 (never) to 5 (always). Scores below 13 suggest possible depressive states, while scores below 20 indicate compromised wellbeing. The total score ranges from 0 to 25, with higher scores indicating a higher level of wellbeing, a score of less than 20 suggests the presence of a depressive disorder, and scores of less than or equal to 13 indicate compromised wellbeing and suggest further analysis for depression. This instrument has a Cronbach’s alpha of.83 (34).
**EuroQol – EQ-5D**: This instrument measures health-related quality of life across five dimensions: mobility, personal care, usual activities, pain/discomfort, and anxiety/depression. A Visual Analog Scale (VAS) is also included to capture participants’ overall health status on a scale of 0 to 100. The value of Cronbach alpha coefficient for EQ-5D was 0.716, which signifies an acceptable internal coherence (35).
**The Barthel Index**: This tool assesses functional independence in daily living activities such as eating, bathing, and mobility. Scores range from 0 (completely dependent) to 20 (fully independent), with the Portuguese version validated in 2007. Instead of the original scale of 0, 5, 10, 15, the validated version uses a scale of 0 to 3 per item. Thus, the total score can vary from 0 to 20 points, where 0 indicates the greatest dependence and 20 the greatest independence. In the validated version, the score is assigned as follows: 0 points for total dependence, 1 point for significant dependence, 2 points for mild dependence and 3 points for total independence. The instrument shows excellent reliability, with a Cronbach’s alpha of 0.96, indicating high internal consistency (36).
**The Lawton and Brody Scale**: This scale evaluates participants’ ability to perform instrumental activities of daily living, such as managing finances, shopping, and using transportation. Higher scores reflect greater autonomy. The Portuguese version, validated by Araújo et al. in 2008, adopts a polytomous score of 0 to 4 for each item, allowing a total assessment of 0 to 32 points, which improves discrimination of the level of (in)dependence. Validation confirmed its suitability for the older adults population in a community setting, with a Cronbach’s alpha of 0.94, indicating high reliability. The correlation with the Barthel Index was significant (*r* = 0.82; *p* ≤ 0.01), demonstrating the relationship between autonomy in IADLs and BADLs (37).
**The UCLA Loneliness Scale**: A 16-item scale used to measure subjective loneliness, with higher scores indicating greater loneliness. The Portuguese version was validated in 2010. In general, the UCLA, Portuguese version, was made up of 16 items, has high consistency and reliability, the scale score varies between 16 and 64 points, with a cut-off point of 32 in the Portuguese population, i.e., older adults people who answer the questionnaire, when the final calculation of the points obtained, show values >32, indicating experiences with negative feelings of Loneliness (38).
**The Health Literacy Population Survey (HLS19-Q12)**: This 12-item tool assesses health literacy, covering health care, disease prevention, and health promotion. Responses are categorized into different literacy levels (excellent, sufficient, problematic, and inadequate). Each answer is assigned a numerical value, where “very easy” receives the highest score and “very difficult” the lowest. These values are added together to generate a total health literacy score for each person. The total score reflects the person’s level of ability to access and understand health information, and make informed decisions about caring for their own health. Based on the total values, participants can be categorized into different levels of health literacy. Cronbach’s alpha coefficients for the HLS19-Q12 typically range from 0.80 to 0.90, with a mean and median of 0.86, indicating high reliability (39).

### 2.6 Data analysis

Data were collected using Google Forms and subsequently exported to Excel for preliminary organization. The dataset was then analyzed using IBM SPSS Statistics 29.0.1.0. Descriptive statistics, such as means, medians, and standard deviations, were calculated for each variable. Graphical representations (e.g., boxplots) were used to illustrate data distributions.

Inferential statistics were used to explore relationships between variables. T-tests, Mann–Whitney U tests, and chi-square tests were employed to examine differences in wellbeing, quality of life, and health literacy between subgroups (e.g., gender, rural vs. urban). Bivariate analyses were conducted to identify significant associations between variables. Results were considered statistically significant when rejected Alpha at *p* < 0.05. Power analysis and sample size calculation were performed in advance using SurveyMonkey software.

## 3 Results

The study involved 344 participants, 52% of whom were female. The participants’ ages ranged from 65 to 96 years, with a mean age of 75.32 years (SD = 7.31). The majority (73%, *n* = 251) were married or in civil partnerships, while 17.2% (*n* = 59) were widowed, 7.6% (*n* = 26) were single, and 2.3% (*n* = 8) were divorced or separated. Most participants (72.1%, *n* = 248) resided in rural areas, and 27.9% (*n* = 96) lived in urban areas. Regarding living arrangements, 68.9% (*n* = 237) lived with their spouse, 18% (*n* = 62) lived alone, 10.5% (*n* = 36) lived with other family members, and 2.6% (*n* = 9) lived with others.

In terms of education, 65.4% (*n* = 225) had completed only primary school, while 13.4% (*n* = 46) were illiterate. A smaller proportion had secondary (11.9%, *n* = 41), higher secondary (3.8%, *n* = 13), or university-level education (2%, *n* = 7). Most participants (92.7%, *n* = 319) were retired, while 5.8% (*n* = 20) were employed, and 1.5% (*n* = 5) were unemployed (Table 1).

**Table 1:** Sociodemographic characteristics of participants.

Variables	n (%)	Chi-Square (χ^2^)	Degrees of freedom (df)	*p*-value
Sex		0.5697674418604651	1	0.45035134358487394
Male	179			
Female	165			
Total	344			
Marital status		437.6511627906977	3	1.5443671559428502e-94
Married	251 (73%)			
Widowed	59 (17.2%)			
Single	26 (7.6%)			
Divorced or separated	8 (2.3%)			
Residence area		67.16279069767442	1	2.4998812545585953e-16
Rural	248 (72.1%)			
Urban	96 (27.9%)			
Living arrangements		369.8372093023256	3	7.550667070893963e-80
With husband or wife	237 (68.9%)			
Alone	62 (18%)			
With other family members	36 (10.5%)			
With others	9 (2.6%)			
Education		490.89156626506025	4	6.251326332358001e-105
Illiterate	46 (13.4%)			
Completed basic education	225 (65.4%)			
Secondary education	41 (119%)			
Higher secondary education	13 (3.8%)			
Higher education	7 (2%)			
Employment status		547.156976744186	2	1.5359325088332778e-119
Retired	319			
Employed	(92.7%)			
Unemployed	20 (5.8%) 5 (1.5%)			

From the Chi-Square Goodness-of-Fit results, several variables demonstrated statistically significant differences (*p* < 0.05). These results suggest substantial variability in the distributions of marital status, residence area, living arrangements, and education, while the distribution of sex remains relatively uniform.

### 3.1 Wellbeing

The WHO-5 Wellbeing Index revealed an average score of 53 (SD = 4.29), indicating generally preserved wellbeing, but with notable variability (Table 2). Gender differences were significant, with men scoring higher on the wellbeing scale (mean = 14.35, SD = 3.32) compared to women (mean = 12.40, SD = 2.79), with a statistically significant difference (*p* < 0.001).

**Table 2:** WHO-5 wellbeing scores by gender and residence.

Question	Average	Standard deviation	Minimum	Maximum
1. I felt happy and in a good mood.	2.76	1.22	0	5
2. I felt calm and peaceful.	2.82	1.23	0	5
3. I felt active and energetic.	2.79	1.19	0	5
4. I woke up feeling fresh and rested.	2.78	1.19	0	5
5. My day-to-day has been filled with things that interest me.	3.05	1.18	0	5
Total	13.20	4.29	3	25

Wellbeing scores were also significantly higher for participants living in urban areas (mean = 14.12, SD = 2.90) compared to those in rural areas (mean = 12.75, SD = 3.21), with a significant Mann–Whitney U test result (*p* < 0.05).

### 3.2 Quality of life

The EQ-5D-3L scale provided insights into participants’ health-related quality of life across five dimensions: mobility, personal care, usual activities, pain/discomfort, and anxiety/depression. Chi-Square Test of Independence was conducted to analyze the association between the levels of problems across the five dimensions of the EQ-5D-3L profile: Mobility, Personal Care, Usual Activities, Pain/Discomfort, and Anxiety/Depression. The contingency table used for the analysis consisted of three levels of problems (“No Problems,” “Some Problems,” and “Severe Problems”) for each of the five dimensions, forming a 5×3 structure. The test results showed a highly significant association between the levels of problems across the dimensions, with χ^2^(8) = 155.37, *p* < 0.001. This indicates that the distribution of responses significantly differs among the dimensions analyzed.

Additionally, the expected frequencies for each cell in the contingency table were calculated to validate the test assumptions. Over 50% reported no issues with mobility and usual activities; however, 41.6% (*n* = 143) reported some problems with mobility, and 34.6% (*n* = 119) with usual activities. Pain and discomfort were prevalent, with 57.3% (*n* = 197) reporting some degree of pain, and 38.7% (*n* = 133) reporting no significant pain. Anxiety and depression affected 48.5% (*n* = 167) of participants, while 49.4% (*n* = 170) reported no issues (Table 3).

**Table 3:** EQ-5D-3L health dimension distribution.

EQ-5D-3L profile	Mobility	Personal care	Usual activities	Pain/discomfort	Anxiety/depression
I have no problems	198 (57.6%)	274 (79.7%)	204 (59.3%)	133 (38.7%)	170 (49.4%)
I have some problems	143 (41.6%)	56 (16.3%)	119 (34.6%)	197 (57.3%)	167 (48.5%)
I have problems	3 (0.9%)	14 (4.1%)	21 (6.1%)	14 (4.1%)	7 (2%)

A Chi-Square Test of Independence was performed to assess the association between gender and mobility levels. The results revealed a statistically significant relationship, with χ^2^(2) = 9.46, *p* = 0.009. This finding indicates that mobility levels vary significantly across genders in the sample. The observed association suggests that gender may play a role in influencing mobility levels. On average, women report lower levels of mobility compared to men. The mean mobility score for women is 1.50, while for men it is 1.35. This indicates that women experience greater challenges with mobility in this sample. A Chi-Square Test of Independence was conducted to assess the association between habitation location (rural or urban) and levels of anxiety or depression. The analysis revealed no statistically significant relationship, with χ^2^ (2) = 0.86, *p* = 0.652. These findings suggest that, within this sample, the location of habitation does not have a significant influence on levels of anxiety or depression.

**Figure 1:**
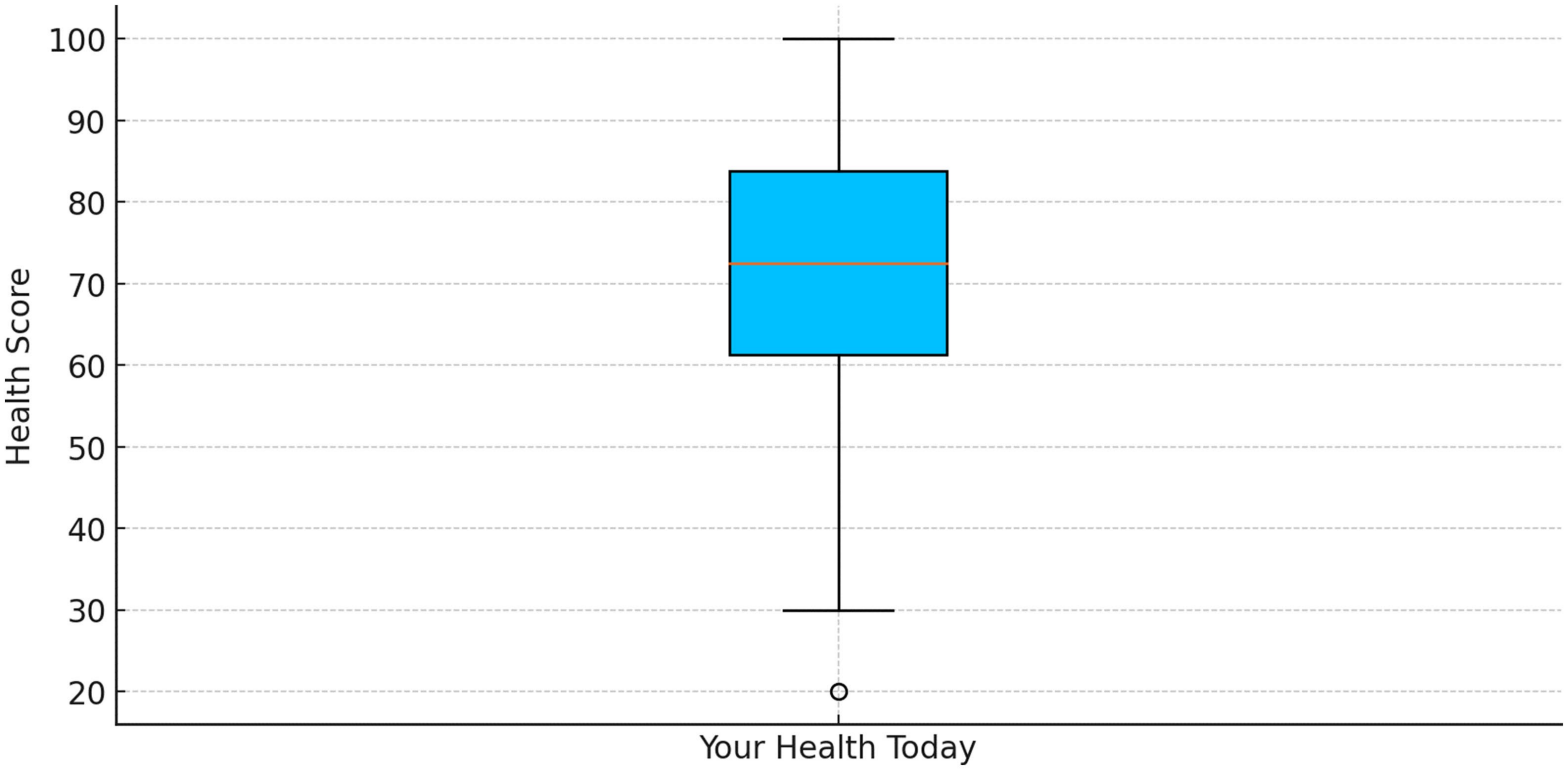
VAS health status scores.

Participants rated their overall health on the EQ-5D VAS with an average score of 68.09 (SD = 12.31) and a median of 70 (Figure 1). Men had significantly higher quality-of-life scores compared to women (*p* < 0.01), with men scoring an average of 1.35 points higher on the overall quality-of-life scale.

### 3.3 Functional assessment

The Barthel Index showed that participants retained a high level of independence in activities of daily living (ADLs), with an average score of 15 (SD = 3.12). The activities with the highest level of independence included “Eating” (mean = 1.91, SD = 0.31), “Using the bathroom” (mean = 1.91, SD = 0.33), and “Bowel control” (mean = 1.94, SD = 0.26). However, dependence was more common in activities such as “Bathing” (mean = 0.88, SD = 0.33) and “Bladder control” (mean = 1.83, SD = 0.41). The most challenging activity was “Climbing stairs” (mean = 1.58, SD = 0.59) (Table 4). The Chi-square goodness-of-fit tests show statistically significant differences (*p* < 0.05) for all activities of daily living (ADLs) in the Barthel Index. This suggests that the observed distribution of scores deviates significantly from an equal distribution, indicating areas where participants’ performance varies.

**Table 4:** Barthel index scores for ADLs.

*N*		Minimum	Maximum	Average	Standard deviation	Chi-square (χ^2^)	*p*-value
Nutrition	344	0	2	1.91	0.312	4063.30	0.00
Dress/undress	344	0	2	1.85	0.390	3561.58	0.00
Bath	344	0	1	0.88	0.328	3709.84	0.00
Personal hygiene	344	0	1	0.97	0.168	4524.72	0.00
Use of the bathroom	344	0	2	1.91	0.329	4036.00	0.00
Intestinal control	344	0	2	1.94	0.263	4274.52	0.00
Bladder control	344	0	2	1.83	0.410	3471.32	0.00
Going up/down stairs	344	0	2	1.58	0.595	2262.95	0.00
Transfer of chair/bed	344	0	3	2.74	0.604	3127.54	0.00
Wandering	344	0	3	2.71	0.573	2868.70	0.00
N valid (from list)	344						

### 3.4 Instrumental activities of daily living

On the Lawton and Brody IADL scale, participants demonstrated strong capacity in areas like “Taking care of the house” (mean = 2.92, SD = 1.25) and “Using the phone” (mean = 2.39, SD = 0.82). The lowest scores were observed in “Doing laundry” (mean = 1.37, SD = 0.70). Variability in abilities was noted, with “House care” showing the most variation across participants (Table 5).

**Table 5:** Lawton and Brody IADL scores.

*N*		Minimum	Maximum	Average	Standard deviation
1. Ability to use a phone	344	0	3	2.39	0.822
2. Shopping	344	0	3	2.25	0.934
3. Food preparation	344	0	3	2.19	0.983
4. Take care of the house	344	0	4	2.92	1.248
5. To do the laundry	344	0	2	1.37	0.704
6. Using means of transportation	344	0	3	2.34	0.968
7. Responsibility with your medication	344	0	2	1.79	0.511
8. Ability to manage your economic affairs	344	0	2	1.63	0.615
N valid (from list)	344				

### 3.5 Loneliness

Loneliness was prevalent, with 50.6% of participants (*n* = 174) scoring higher than 32 on the UCLA Loneliness Scale, indicating significant feelings of loneliness (mean score = 41.93, SD = 5.59). The remaining 49.4% (*n* = 170) had scores of 32 or below, indicating no significant loneliness (mean score = 24.65, SD = 4.89).

Women were more likely to experience loneliness than men, with a significant association between gender and loneliness (*p* < 0.05). The observed count of older adults people with loneliness was higher among women (*n* = 103) than among men (*n* = 71). Loneliness was also more prevalent among rural residents (*n* = 136) compared to urban residents (*n* = 38), with a statistically significant association between place of residence and loneliness (*p* < 0.05) (Table 6).

**Table 6:** UCLA loneliness scale scores by gender and residence.

*N*		Minimum	Maximum	Average	Standard deviation
Loneliness>32	174	33	57	41.93	5.589
Without loneliness≤32	170	16	32	24.65	4.896
N valid (from list)	0				

### 3.6 Health literacy

**Figure 2:**
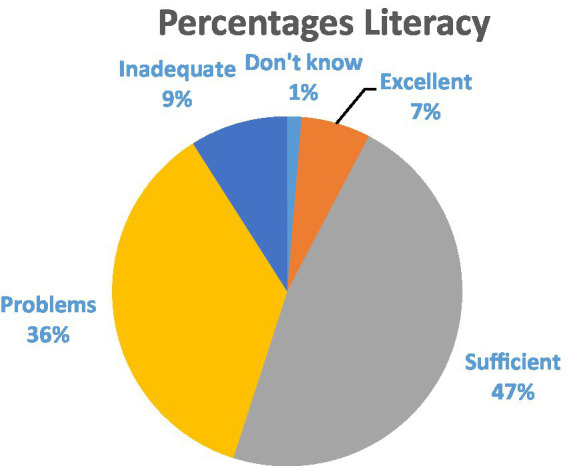
Results of the Health Literacy Population Survey in percentages.

Health literacy results from the HLS19-Q12-pt indicated that only 6.39% of participants (*n* = 22) demonstrated excellent literacy, while 47.27% (*n* = 163) had sufficient literacy. Problematic literacy was found in 35.98% (*n* = 124) of participants, and 9.03% (*n* = 31) exhibited inadequate literacy (Figure 2). There was no significant association between gender and health literacy (*p* > 0.05). However, rural residents had significantly lower health literacy than urban residents (*p* < 0.05), indicating geographical disparities in health knowledge.

## 4 Discussion

This study aimed to assess the quality of life, wellbeing, functionality, loneliness, and health literacy of older adults individuals in the Alentejo region, revealing key differences based on gender and geographical location that are crucial for informing future interventions.

The sociodemographic analysis of the participants revealed the diversity of the sample and the impact that contextual factors can have on the population’s wellbeing and quality of life. The sample is balanced in terms of gender (52% female and 48% male), with ages ranging from 65 to 96, reflecting an aging population with an average age of 75.32. This is in line with the National Statistics Institute (2), which states that this area of the country is one of the oldest, where 27% of the inhabitants are aged 65 or over. The majority of this population is married or cohabiting (73%) and lives predominantly in rural areas (72.1%). This distribution suggests that the majority of participants live in potentially more isolated and traditional environments, which may influence their experiences of quality of life, loneliness and literacy, while there is still insufficient research into the experience of older adults people living in different living environments: rural–urban (27).

### 4.1 Wellbeing and quality of life

The findings indicate that while wellbeing is generally preserved among the older adults participants, there are significant disparities between men and women, as well as between urban and rural residents. Men reported higher wellbeing scores than women, a trend that aligns with previous research suggesting that older women are more vulnerable to emotional distress, possibly due to caregiving responsibilities, limited social engagement, and increased physical limitations in later life (29, 40). In particular, older women may benefit from social and recreational programs aimed at improving emotional wellbeing (41).

Similarly, rural residents reported lower wellbeing compared to urban residents, likely due to reduced access to healthcare services, social activities, and transportation (3). Although rural environments can offer benefits such as green spaces that contribute to health, the limited access to services in these areas poses a challenge. Improving transportation, healthcare access, and community engagement opportunities in rural areas could mitigate these issues (3, 42, 43).

In terms of quality of life, the EQ-5D-3L results revealed that mobility issues, pain/discomfort, and anxiety/depression were significant challenges for many participants, particularly in rural areas. These issues negatively impact quality of life and highlight the need for targeted interventions, such as pain management programs, mental health services, and mobility support (44, 45). Enhancing healthcare infrastructure in rural areas is crucial to addressing these challenges.

### 4.2 Functionality and autonomy

The high scores on the Barthel Index suggest that participants maintain significant independence in basic activities of daily living (ADLs), although mobility-related activities, such as climbing stairs, pose more difficulty. As physical decline is a natural part of aging, these results underscore the importance of interventions like physical therapy and rehabilitation programs that help older adults individuals maintain their independence (45).

Challenges were also evident in more complex instrumental activities of daily living (IADLs), such as house care and laundry. These findings are consistent with the literature, which shows that older adults often struggle with more demanding tasks (37). Community-based services that offer domestic support or transportation could enhance the independence and autonomy of older adults individuals.

### 4.3 Loneliness, gender and location

The study revealed that over half of the participants experienced significant feelings of loneliness, with women and rural residents being particularly affected. Loneliness is a well-documented risk factor for mental and physical health problems in older adults, including depression and cardiovascular issues, and is linked to increased mortality rates (15–17). The greater prevalence of loneliness in rural areas is consistent with other findings, where geographical isolation reduces social opportunities (30, 43, 46).

Social prescription programs, which connect individuals with community-based activities, may be effective in alleviating loneliness, particularly in rural settings (47–49). Additionally, promoting digital literacy among older adults could provide alternative ways to maintain social connections, even in geographically isolated areas (25).

### 4.4 Health literacy

Low levels of health literacy were a significant concern in this study, with nearly half of the participants displaying problematic or inadequate literacy. This finding aligns with previous research showing that older adults, particularly those in rural areas, often struggle to understand health-related information, which can hinder effective health management (19–21).

Improving health literacy is essential for empowering older adults to manage their health more effectively. Tailored educational programs, simplified health communication, and the use of digital tools could help close the health literacy gap, especially in rural areas (22–25).

### 4.5 Recommendations for community interventions in rural regions

Based on the results of this study and existing literature, it is clear that community interventions play a crucial role in promoting active and healthy aging in rural regions. To achieve this goal, specific strategies must be implemented, including social prescribing, which is emerging as a promising approach to meeting the physical, social, and emotional needs of older adults (50). Social prescribing is an innovative practice that involves directing older adults to community activities and resources that promote health and wellbeing. Examples include participation in support groups, exercise classes, recreational activities, and other services offered by the community (47). These initiatives have been shown to be effective in increasing social engagement, promoting mental and physical health, and significantly reducing loneliness. Studies indicate that social prescription positively impacts the quality of life of older adults, connecting them to community support networks and significant opportunities for social interaction (48, 49).

## 5 Conclusion

This study provides insights into the wellbeing, quality of life, functionality, loneliness, and health literacy of older adults individuals in the Alentejo region. The findings highlight significant disparities based on gender and geographical location, with women and rural residents particularly vulnerable to lower wellbeing, higher levels of loneliness, and poorer health literacy.

To address these issues, the results of this study, complemented with the known reality of frequent contact with users, highlights that public health strategies must prioritize improving access to health care and social services in rural areas. Specific attention should be given to managing mobility issues, pain, and mental health conditions like anxiety and depression. Furthermore, enhancing health literacy through educational programs and simplifying medical information are essential to empower older adults to take control of their health.

Social prescribing and community-based initiatives that aim to reduce loneliness could significantly improve the quality of life for older adults individuals, especially those living in rural areas. Such programs should be designed with a focus on accessibility for rural residents and should promote digital literacy to help maintain social connections.

### 5.1 Implications for policy and practice

The results of this study underscore the need for public health interventions that address the specific challenges faced by the older adults population in Alentejo, particularly women and rural residents.

To ensure healthy and active aging for the older adults in Alentejo and similar regions, the following public health policies should be prioritized:

- Healthcare Access: Improve healthcare services in rural areas through mobile health units, telemedicine, and transportation support.
- Loneliness Reduction: Implement social programs aimed at reducing loneliness, such as community centers, digital literacy training, and support groups.
- Health Literacy: Enhance health literacy by creating easy-to-understand health resources and educational programs tailored to older adults, particularly in rural areas.
- Gender-sensitive Interventions: Develop interventions focused on supporting women, especially those who experience social isolation or have caregiving responsibilities.

### 5.2 Future research

Future studies should focus on longitudinal research to explore how these variables change over time and how effective interventions are in improving wellbeing and quality of life. Additional research is also needed to examine the specific challenges faced by older adults individuals in more remote areas and the role of emerging technologies, such as telemedicine, in improving access to care and social engagement.

### 5.3 Limitations

While this study offers insights, it is necessary to acknowledge certain limitations. The study utilized a non-probabilistic convenience sampling method, involving users of the health unit where one of the researchers is employed, with authorization from the Ethics Committee limited to that specific unit. This may restrict the generalizability of the findings to the broader older adults population. Calculations were performed, allowing for a margin of error, to ensure that the sample was representative of the health unit’s population. Additionally, the cross-sectional design only provides a snapshot of participants’ conditions, meaning causal relationships cannot be inferred.

Despite these limitations, the study is a contribution to understanding the challenges faced by older adults individuals in rural areas, particularly in terms of health and social needs. The findings offer practical recommendations for promoting healthy aging in the Alentejo region and beyond.

## Data Availability

The original contributions presented in the study are included in the article/supplementary material, further inquiries can be directed to the corresponding author.

## References

[1] United Nations Department of Economic and Social Affairs. World population ageing 2019. United Nations. (2019). Available online at: https://digitallibrary.un.org/record/3846855

[2] INE. Censos 2021 - Divulgação dos Resultados Provisórios. Inst Nac Estatística Censos 2021- Divulg dos Result Provisórios. (2021). Available online at: https://www.ine.pt/xportal/xmain?xpid=INE&xpgid=ine_destaques&DESTAQUESdest_boui=526271534&DESTAQUESmodo=2

[3] Comissão Económica das Nações Unidas para a Europa. Resumo de políticas da Unece sobre o envelhecimento nº 18.Idosos em áreas rurais e remotas. (2017). 1–31.

[4] Hong A Sallis JF King AC Conway TL Saelens B Cain KL. Linking green space to neighborhood social capital in older adults: the role of perceived safety. Soc Sci Med. (2018) 207:38–45. doi: 10.1016/j.socscimed.2018.04.051, PMID: 29727748

[5] Tabrizi N Lak A Moussavi ASMR. Green space and the health of the older adult during pandemics: a narrative review on the experience of COVID-19. Front Public Heal. (2023) 11:1218091. doi: 10.3389/fpubh.2023.1218091, PMID: 37601191 PMC10433209

[6] World Health Organization. Regional Office for Europe. Action plan for the prevention and control of noncommunicable diseases in the WHO European region. World Heal Organ Eur. (2016). Available online at: https://apps.who.int/iris/handle/10665/341522?show=full

[7] Lloyd-Sherlock P Beard J Minicuci N Ebrahim S Chatterji S. Hypertension among older adults in lowand middle-income countries: prevalence, awareness and control. Int J Epidemiol. (2014) 43:116–28. doi: 10.1093/ije/dyt215, PMID: 24505082 PMC3937973

[8] Cheng W Song W Ye C Wang Z. Family networks, social networks, and life satisfaction of older adults in China. Healthcare. (2022) 10:1–18. doi: 10.3390/healthcare10081568, PMID: 36011225 PMC9407943

[9] INE IN de E. Projeções de População Residente 2080. Contudo, na Área Metropolitana de Lisboa e no Algarve a população residente poderá aumentar. Destaque informação à Comun Soc. (2020):1–21.

[10] García LMR Ramírez Navarrro JM. The impact of quality of life on the health of older people from a multidimensional perspective. J Aging Res. (2018) 2018:1–7. doi: 10.1155/2018/4086294, PMID: 29888005 PMC5977005

[11] Finance Personal Research Centre. Financial wellbeing in later life. Univ Bristol. (2014)

[12] Chen J Liu L Shea JL. The impact of positive self-perceptions of aging on subjective well-being through the mediation of psychological resilience among community-dwelling older adults during COVID-19 in Taiwan. Health Soc Care Commun. (2024) 2024:4755146

[13] Liu J Kwan C Deng JHY. The mental health impact of the COVID-19 pandemic on older adults in China: a systematic review. Int J Env Res Public Heal. (2022) 19:14362. doi: 10.3390/ijerph192114362, PMID: 36361241 PMC9657377

[14] Lebrasseur A Fortin-Bédard N Lettre J Raymond E Bussières EL Lapierre N. Impact of the COVID-19 pandemic on older adults: rapid review. JMIR Aging. (2021) 4:1–17. 10.2196/26474 PMC8043147 33720839

[15] Kyriazis M Mikellides G Pantelidakis H Polycarpou M Panayiotou B. COVID-19 isolation and risk of death in Cyprus elderly people. Front Med. (2021) 8:717692. doi: 10.3389/fmed.2021.717692, PMID: 34409055 PMC8365165

[16] Kim JE Lee YL Chung MA Yoon HJ Shin DE Choi JH. Effects of social prescribing pilot project for the elderly in rural area of South Korea during COVID-19 pandemic. Heal Sci Rep. (2021) 4:1–11. doi: 10.1002/hsr2.320, PMID: 34250272 PMC8247938

[17] Holt-lunstad J Smith TB. Loneliness and social isolation as risk factors for mortality: a meta-analytic review. Perspect Psychol Sci. (2015) 10:227–37. doi: 10.1177/1745691614568352 25910392

[18] Fakoya OA Mccorry NK Donnelly M. Loneliness and social isolation interventions for older adults: a scoping review of reviews. BMC Public Health. (2020) 20:129. doi: 10.1186/s12889-020-8251-6, PMID: 32054474 PMC7020371

[19] Costa A Feteira-santos R Alarc V Henriques A. Health literacy among older adults in Portugal and associated sociodemographic, health and healthcare-related factors. Int J Environ Res Public Health. (2023) 20:4172. doi: 10.3390/ijerph20054172 36901182 PMC10002045

[20] Chiu H Tsai H Kuo KN Leung AYM Chang Y. Exploring the influencing factors of health literacy among older adults: a cross-sectional survey. J Med. (2020) 56:1–12. 10.3390/medicina56070330 PMC7404792 32630726

[21] Tao S Sun S Wu S Peng T Cao L Yan M. Current status and influencing factors of health literacy among older adults in combined medical and nursing care institutions: a cross-sectional study. Front Med. (2024) 11:4. doi: 10.3389/fpubh.2023.1323335, PMID: 38292383 PMC10825950

[22] Chesser AK Woods NK Smothers K Rogers N. Health literacy and older adults: a systematic review. Gerontol Geriatr Med. (2016) 2:2333721416630492. doi: 10.1177/2333721416630492 28138488 PMC5119904

[23] Kobayashi LC Wardle J Von WC. Limited health literacy is a barrier to colorectal cancer screening in England: evidence from the English longitudinal study of ageing. Prev Med. (2014) 61:100–5. doi: 10.1016/j.ypmed.2013.11.012, PMID: 24287122 PMC3969575

[24] Kang H Baek J Chu SH Choi J. Digital literacy among Korean older adults. Digit Health: a scoping review of quantitative studies. (2023). doi: 10.1177/20552076231197334 PMC10467254 37654708

[25] Tomczyk Ł Mascia ML Gierszewski D Walker C. Barriers to digital inclusion among older people: a intergenerational reflection on the need to develop digital competences for the group with the highest level of digital exclusion. Innoeduca Int J Technol Educ Innov. (2023) 9:5–26.

[26] Hussain B Mirza M Baines R Burns L Stevens S Asthana S. Loneliness and social networks of older adults in rural communities: a narrative synthesis systematic review. Front Public Health. (2023) 11:1113864. doi: 10.3389/fpubh.2023.1113864, PMID: 37255758 PMC10225733

[27] Rey-Beiro S Martínez-Roget F. Rural-urban differences in older adults’ life satisfaction and its determining factors. Heliyon. (2024) 10:e30842. 38774093 10.1016/j.heliyon.2024.e30842 PMC11107240

[28] Seino S Tomine Y Nishi M Hata T Fujiwara Y Shinkai S. Effectiveness of a community-wide intervention for population-level frailty and functional health in older adults: a 2-year cluster nonrandomized controlled trial. Prev Med. (2021) 149:106620. doi: 10.1016/j.ypmed.2021.106620, PMID: 33992656

[29] Roncon J Lima S Pereira MG. Qualidade De Vida, Morbilidade Psicológica e stress familiar em Idosos Residentes na Comunidade quality of life, psychological morbidity and family stress in elderly residing in the community. Psicol Teor e Pesqui. (2015) 31:87–96. doi: 10.1590/0102-37722015011637087096

[30] Lorenzo Ó Teixeira A Santos S Teixeira D Penaforte H Sequeira C. Fatores de Isolamento Social do Idoso em Meio Rural. Rev Investig Inovação em Saúde. (2019) 2:39–46. doi: 10.37914/riis.v2i2.57

[31] Oliveira TC Bayer S Gonçalves L Barlow J. Telemedicine in Alentejo. Telemed e-Health. (2014) 20:90–3. doi: 10.1089/tmj.2012.0308, PMID: 24180419 PMC3880063

[32] Bonita R Beaglehole R. Basic epidemiology. World Heal Organ. (2006)

[33] Fortin M Côté J Filion F. Fundamentos e etapas do processo de Investigação. Lusodidacta: Publicação (2009).

[34] World Health Organisation. Wellbeing measures in primary health care/the Depcare project. Rep a WHO Meet. (1998). Available online at: http://www.euro.who.int/__data/assets/pdf_file/0016/130750/E60246.pdf

[35] Ferreira PL Ferreira LN Pereira LN. Contribution for the validation of the Portuguese version of EQ-5D contributos Para a Validação da Versão Portuguesa do EQ-5D. Acta Med Port. (2013) 26:664–75. doi: 10.20344/amp.1317, PMID: 24388252

[36] Araújo F Oliveira A Pinto C Ribeiro J. Validação do Índice de Barthel numa amostra de idosos não institucionalizados. Rev Port Saúde Pública. (2007) 25:59–66.

[37] Araújo Fátima Pais José L Ribeiro António Oliveira Cristina Pinto TM. Validação da escala de Lawton e Brody numa amostra de idosos não institucionalizados. Actas do 7^o^ Congr Nac Psicol da saúde. (eds) Leal I Pais-Ribeiro J Silva I Marques I. Lisboa, PT: ISPA. (2007):217–220.

[38] Pocinho MTS Farate C Dias CA. Validação Psicométrica da Escala UCLA-Loneliness para Idosos Portugueses. Interações Soc e novas Mod. (2010) 18:65–77.

[39] WHO. The HLS 19-Q12 instrument to measure general health literacy development of the instrument. Action Netw Meas Popul Organ Heal Lit. (2022) June:2–9.

[40] Oliveira GPL Victória Rocha dos Santosb ASL Karoline Alves Correa Boscalhad LCMS Pampolimf G. A influência do gênero nos domínios da qualidade de vida em idosos de uma unidade de saúde de V itória-ES The influence of gender on quality of life in elderly people at a health unit in V itória-ES. Clin Biopsychosoc. (2023) 1:40–5.

[41] Shi X Li Y Sun L Yu Y. Gender differences in the subjective well-being of older adult learners in China. Front Psychol. (2022) 13:1043420. doi: 10.3389/fpsyg.2022.1043420, PMID: 36438379 PMC9683339

[42] Paúl C Fonseca A Martín JA. Satisfação e Qualidade de Vida em idosos portugueses In: Envelhecer em Portugual Psicologia, saúde e prestação de cuidados. Lisboa: CLIMEPSI Editores (2005). 75–95.

[43] van Hoof J Marston HR Kazak JK Buffel T. Ten questions concerning age-friendly cities and communities and the built environment. Build Environ. (2021) 199:107922. doi: 10.1016/j.buildenv.2021.107922, PMID: 40182660

[44] Shafrin J Sullivan J Goldman DP Gill TM. The association between observed mobility and quality of life in the near elderly. PLoS One. (2017) 12:e0182920. doi: 10.1371/journal.pone.0182920, PMID: 28827806 PMC5572211

[45] Noto S. Perspectives on aging and quality of life. Healthcare (Basel) (2023) 11:2131. doi: 10.3390/healthcare11152131 PMC10418952 37570372

[46] Byrne KA Anaraky RG Dye C Ross LA Madathil KC Knijnenburg B. Examining rural and racial disparities in the relationship between loneliness and social technology use among older adults. Front Public Health. (2021) 9:723925. doi: 10.3389/fpubh.2021.723925, PMID: 34532308 PMC8438168

[47] Mendes A. Social prescribing in the community. Br J Community Nurs. (2021) 26:204–5. doi: 10.12968/bjcn.2021.26.4.204, PMID: 33797961

[48] Colucci E Nadeau S Higgins J Kehayia E Poldma T Saj A. Since January 2020 Elsevier has created a COVID-19 resource centre with free information in English and mandarin on the novel coronavirus COVID-19. The COVID-19 resource centre is hosted on Elsevier Connect, the company’ s public news and information. (2020) 99:104606.

[49] Islam MM. Social prescribing—an effort to apply a common knowledge: impelling forces and challenges. Front Public Heal. (2020) 8:515469. doi: 10.3389/fpubh.2020.515469, PMID: 33330299 PMC7728793

[50] MacLeod S Schwebke K Hawkins K Ruiz J Hoo E Yeh CS. Need for comprehensive health care quality measures for older adults. Popul Health Manag. (2018) 21:296–302. doi: 10.1089/pop.2017.0109, PMID: 29064345 PMC6070128

